# Extraction of S- and N-Compounds from the Mixture of Hydrocarbons by Ionic Liquids as Selective Solvents

**DOI:** 10.1155/2013/512953

**Published:** 2013-06-12

**Authors:** Beata Gabrić, Aleksandra Sander, Marina Cvjetko Bubalo, Dejan Macut

**Affiliations:** ^1^INA-Industrija Nafte d.d., Avenija Veceslava Holjevca 10, 10000 Zagreb, Croatia; ^2^Faculty of Chemical Engineering and Technology, University of Zagreb, Marulićev Trg 19, 10000 Zagreb, Croatia; ^3^Faculty of Food Technology and Biotechnology, University of Zagreb, Pierottijeva 6, 10000 Zagreb, Croatia

## Abstract

Liquid-liquid extraction is an alternative method that can be used for desulfurization and denitrification of gasoline and diesel fuels. Recent approaches employ different ionic liquids as selective solvents, due to their general immiscibility with gasoline and diesel, negligible vapor pressure, and high selectivity to sulfur- and nitrogen-containing compounds. For that reason, five imidazolium-based ionic liquids and one pyridinium-based ionic liquid were selected for extraction of thiophene, dibenzothiophene, and pyridine from two model solutions. The influences of hydrodynamic conditions, mass ratio, and number of stages were investigated. Increasing the mass ratio of ionic liquid/model fuel and multistage extraction promotes the desulfurization and denitrification abilities of the examined ionic liquids. All selected ionic liquids can be reused and regenerated by means of vacuum evaporation.

## 1. Introduction

Diesel and gasoline rich in sulfur will produce exhaust gases containing SO_x_, a major contributor to air pollution and acid rain. Due to strict regulatory requirements on sulfur content in fuels, this problem is widely investigated [1–15]. 

Today, a commonly used industrial process for desulfurization of diesel and gasoline is hydrodesulphurization (HDS), in which organic sulfur compounds are converted to H_2_S and the corresponding hydrocarbons. The process requires high temperatures and pressures, as well as large amounts of hydrogen, making HDS an expensive and relatively environmentally unfriendly process. Moreover, this process requires even more expensive process conditions for removal of compounds such as dibenzothiophene derivatives [1–4, 7]. For that reason, scientists explore new environmentally friendly and energy-saving processes [1, 5].

Liquid-liquid extraction is one of such processes. The major advantage of extraction is in its mild operating conditions (low temperatures and pressures—ambient conditions) and consequently conservation of significant amount of energy. As for any other process that involves mass-separating agent, special care must be taken when selecting a proper solvent [16]. However, liquid-liquid extraction generates organic waste which requires disposal. In order to minimize that waste, the solvent should be recyclable, reusable, and regenerable [4–6, 13, 14, 16, 17]. Solvents commonly used in industry are volatile organic compounds that exhibit high-vapor pressure. Additionally, volatile organic solvents are flammable and are of varying toxicity, depending on their nature, so the major task of many researchers is to find a way of replacing VOCs with environmentally friendly, so-called *green solvents* [18]. This is not a simple problem, because one cannot simply replace one solvent with another. One possible solution is replacement of organic solvents with ionic liquids. Ionic liquids are very good solvents for a wide range of organic, inorganic, and polymeric compounds. Owing to their negligible vapor pressure, ionic liquids are considered as *green solvents*. But even if ionic liquids are not responsible for air pollution, their impact on complete ecosystem is still unexplored. For instance, many ionic liquids are soluble in water, so they can enter the environment by this path [1].

During the past decade, an increasing trend of investigations concerning the possibilities of replacing the existing desulfurisation processes with the extraction of fuels by ecologically acceptable ionic liquids can be observed. Authors mostly use model solutions of different compositions as the representatives of real diesel and gasoline [3, 5, 6, 8–11, 13, 15, 16] as well as real refinery samples [2, 4, 7, 8, 15, 16]. Most commonly used are the imidazolium-based ionic liquids with 1-alkyl-3-methylimidazolium cations ([C*n*C1Im]) and various anions. The use of the pyridinium-based ionic liquids for desulfurization of diesel and gasoline was also investigated [7, 14, 16]. Denitrogenation of feeds before HDS was also investigated in order to increase the efficiency of HDS process because N-compounds compete with S-compounds on the catalyst surface [19]. Pyridinium and imidazolium ionic liquids were tested, and it was observed that the same ionic liquids have extraction ability to both S- and N-compounds [1, 20, 21].

The aim of this paper is to investigate the applicability of six different ionic liquids for the separation of sulfur and nitrogen compounds from model diesel and FCC gasoline by means of liquid-liquid extraction. The time needed to reach equilibrium in a batch extractor equipped with the mechanical stirrer was also determined. The influences of the hydrodynamic conditions, the mass ratio (ionic liquid/model solution), and the number of extraction stages on the extraction effectiveness were investigated. In addition, the reusability of the selected ionic liquids and regeneration by means of vacuum evaporation was tested.

## 2. Materials and Methods

### 2.1. Chemicals

The list of chemicals used for preparation of model solutions as representatives of FCC gasoline and diesel is as follows: *n*-hexane, *n*-heptane, toluene, and pyridine were purchased from Carlo Erba Reagenti; thiophene, *n*-hexadecane, and dibenzothiophene were purchased from Acros Organics; *n*-dodecane was purchased from Fisher UK and isooctane from Kemika.

The list of chemicals used in the synthesis of ionic liquids is as follows: 3,5-lutidine, 1-bromohexane, and lithium bis(trifluoromethanesulfon)imide were purchased from Acros Organics; 1-methylimidazole was purchased from Alfa Aesar; 1,2-dimethylimidazole, 1-bromopentane, 1-bromoheptane, 1-bromodecane, and benzyl chloride were purchased from Sigma-Aldrich; acetonitrile was purchased from J.T. Baker, ethyl acetate and toluene from Kemika and dichloro methane from TTT Ltd.

1-Ethyl-3-methylimidazolium ethylsulfate was purchased from MERCK.

### 2.2. Model Fuels

Composition of model fuels is presented in Table 1. Representatives of FCC gasoline and diesel are prepared according to the literature [21].

### 2.3. General Procedure for the Preparation of Ionic Liquids

Quaternization of heterocyclic imidazolium or pyridinium compound followed by the anion metathesis was performed according to standard procedures [22–24] or their modifications. Aliphatic halide (1-bromopentane, 1-bromohexane, 1-bromoheptane, or 1-bromodecane) was added in 10% excess to the stirred solution of heterocyclic imidazolium or pyridinium compound (1-methylimidazole, 1,2-dimethylimidazole, or 3,5-dimethylpyridine) in toluene at 0°C, and the reaction mixture was stirred 24–48 h at 70°C. Sedimented quaternary imidazolium or pyridinium halide was washed thoroughly with ethyl-acetate and dried under reduced pressure. The anion metathesis was performed by the treatment of the aqueous solution of obtained halides with lithium bis(trifluoromethanesulfonyl)imide (LiTf_2_N) in excess of 10% and was stirred for approximately 2 h. The upper aqueous phase was decanted, and the lower product portion was washed with water until chloride free, as determined by silver nitrate test. All ILs were dried under high vacuum at 90°C for 8 h prior to use and were characterized by ^1^H NMR.

^1^H NMR spectra were recorded in DMSO-d_6_ on a Bruker AV300 (300 MHz) spectrometer at Ruđer Bošković Institute (Zagreb, Croatia). Chemical shifts were expressed in ppm values using TMS as an internal standard.

The selected ionic liquids are presented in Table 2.

### 2.4. Measurement of Density, Viscosity, Surface Tension, and Interfacial Tension

Viscosity of ionic liquids and model solutions were measured on the Brookfield DV-III Ultra Programmable Rheometer.

Density, surface tension, and interfacial tension were measured on the KRUSS K9 tensiometer.

All measurements were performed at the room conditions (25°C).

### 2.5. Liquid-Liquid Extraction

Liquid-liquid extraction experiments were carried out in a laboratory batch extractor (mixing vessel) equipped with the mechanical stirrer. Quantities of solvent and feed solution were mixed together at the defined mass ratio (ionic liquid/model solution) for a given period of time. The mixing rate was set to the value at which complete dispersion is achieved. This state was visually observed. All systems were mixed with the mixing rate of 500 rpm. Afterward, the heavier and lighter phases are separated in a settling unit. 

Experiments have been performed at four mass ratios of ionic liquid to model solutions (0.25, 0.50, 0.75, and 1.00).

Mass ratio of ionic liquid and model solution was calculated by

(1) $$\begin{array}{c} S=\frac{m\left(\text{ionic liquid}\right)}{m\left(\text{model solution}\right)}. \end{array}$$

Extraction efficiency was calculated by the following equation:

(2) $$\begin{array}{c} \varepsilon =\frac{x_{F}-x_{R}}{x_{F}}. \end{array}$$

The influence of mixing intensity (100–500 rpm) and the number of stages were also investigated. These experiments were performed at the lowest mass ratio (ionic liquid/model solution), *S* = 0.25.

### 2.6. Gas Chromatography

Concentration of all components was determined by means of gas chromatography. The GC used was a GC-2014-Shimadzu equipped with an autosampler, FID detector, and fused silica capillary column CBP1-S25-050 (length: 25 m, inner diameter: 0.32 mm). The GC program parameters for the analyses of both model solutions are presented in the Supplementary Material (see the table in Supplementary Material available online at http://dx.doi.org/10.1155/2013/512953.

Calibration curves were measured and incorporated in the software (Shimadzu GC Solutions), so after analysis mass fractions of all components were calculated automatically.

### 2.7. Regeneration of Ionic Liquids

Due to the negligible vapor pressure of ionic liquids and volatility of components present in ionic liquid after extraction, as a recovery method a vacuum evaporation was selected. For that purpose, an IKA rotary evaporator equipped with the vacuum pump was used. All ILs were dried under high vacuum at 80°C for 8 h. After evaporation, the purity of ionic liquids was determined by means of ^1^H NMR spectroscopy.

## 3. Results and Discussion

The aim of this paper was to select appropriate ionic liquids for desulfurization and denitrification of model gasoline and diesel fuel. As previously mentioned, five of the selected ionic liquids have imidazolium-based cation, and the last one has pyridinium-based cation. Imidazolium-based ionic liquids mostly have the same anion, bis(trifluoromethylsulfonyl)imide. 1-Ethyl-3-methyl imidazolium ethyl sulfate was selected from the literature since, according to the literature cited [21], this ionic liquid is immiscible with the prepared model solutions and real samples. The same statement holds for the pyridinium-based ionic liquid, 1-hexil-3-methylpyridinium bis(trifluoromethylsulfonyl)imide. All ionic liquids were synthesized, except the commercially available [C_2_mim][EtSO_4_].

### 3.1. Physical Properties of Model Solutions and Ionic Liquids

Besides the distribution coefficient, selectivity, mutual solubility of solvents, regeneration, and physical properties of both phases (such as density, viscosity, and interfacial tension) will influence the selection of the most appropriate solvent for liquid-liquid extraction. For the investigated model solutions and ionic liquids, measured results were presented in Table 3. It can be seen that differences in properties between the model solutions and ionic liquids were satisfactory. From the viscosity point of view, the most suitable ionic liquid is [C_5_mim][Tf_2_N] due to the lowest viscosity. The same ionic liquid should be the most appropriate from the interfacial tension point of view. Liquid with lower viscosity and interfacial tension will be easily dispersed into the other, and higher rates for mass transfer will be achieved. The reader should have in mind that the final conclusion about the rate of mass transfer, the solubility of all components of the model solution, the distribution coefficients, and, selectivity must be determined.

### 3.2. Determination of Extraction Time

**Figure 1:**
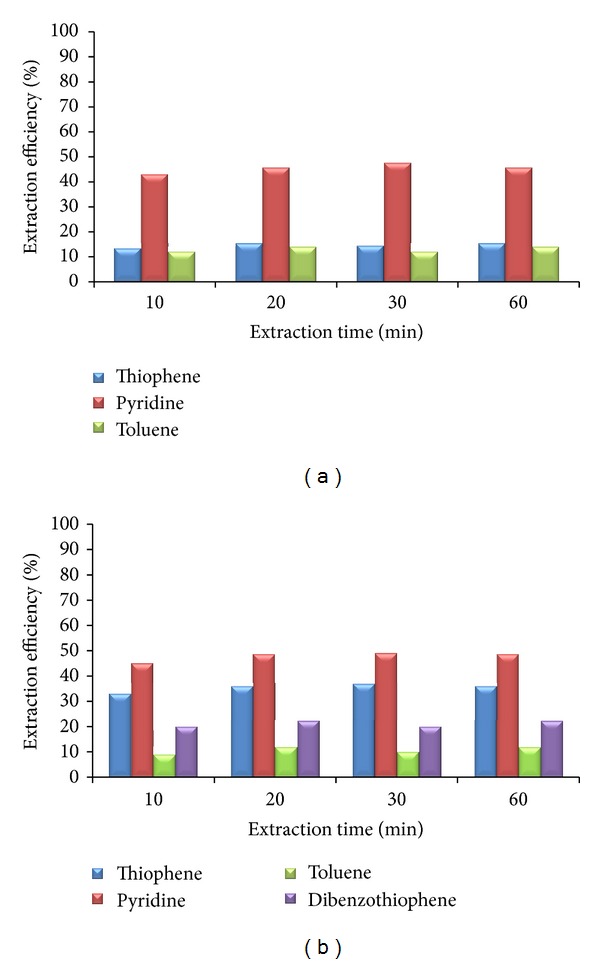
The influence of extraction time on the extraction efficiency ([C_5_mim][Tf_2_N]; *S* = 0,25).

In order to determine the time needed for maximum extraction efficiency, extraction experiments were stopped in defined time intervals. Experiments were performed at the mass ratio of ionic liquid and model solution equal to 0.25. After 10 minutes during which the phases were separated, very small amount of model solution was taken, and the concentration was measured. The procedure was taken from the literature data [2]. With each model solution, experiments have been performed with all ionic liquids. Results obtained after extraction with [C_5_mim][Tf_2_N] for both model solutions are presented in Figure 1. For all key components (thiophene, pyridine, and dibenzothiophene), the maximum extraction efficiency was achieved after 20 minutes. All other ionic liquids act similarly, so extraction time was set to 20 minutes.

### 3.3. The Influence of the Hydrodynamic Conditions

**Figure 2:**
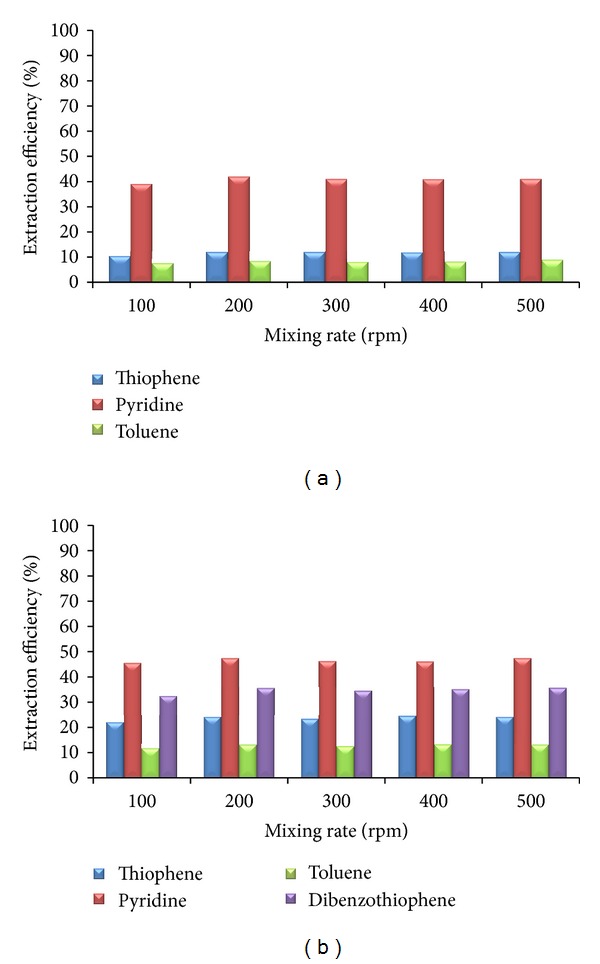
The influence of the mixing intensity on the extraction efficiency ([C_6_mmPy][Tf_2_N]; *S* = 0,25; *t* = 20 min): (a) model solution 1; (b) model solution 2.

In order to see whether the hydrodynamic conditions influence the mass transfer during extraction of S- and N-compounds from the mixture of hydrocarbons with the selected ionic liquids, the experiments have been performed at different mixing intensities. It is well known that higher mixing intensities will result in enhanced mass transfer. At higher mixing rate, resistances to mass transfer are lower, and larger mass transfer area is produced (smaller droplets). The mixing rate was changed from 100 to 500 rpm. The Reynolds number ranges from 1705 for [C_7_mmim][Tf_2_N] (at 100 rpm) up to 9594 for [bzmim][Tf_2_N] (at 500 rpm), for model solution that represents FCC gasoline. For model solution that represents diesel fuel, the Reynolds number ranges from 536 for [C_7_mmim][Tf_2_N] (at 100 rpm) up to 3030 for [bzmim][Tf_2_N] (at 500 rpm). Based on the obtained results, it can be stated that the hydrodynamic conditions do not influence the extraction efficiency of the selected ionic liquids (Figure 2). Physical properties of the involved phases and solubility of key components play a huge role in the transfer of key components between the model solution and selected ionic liquids. The rate of mass transfer is very high, so equilibrium was achieved after short time (Figure 1), even at the lowest mass transfer area (at 100 rpm). High rates of different processes and chemical reactions were reported in the literature [25], as one of the advantages of ionic liquids. Another advantage that is confirmed by our results is high solubility of S- and N-compounds in ionic liquids. For all other ionic liquids and model solutions, the maximum extraction efficiency was also obtained at the lowest mixing rate.

### 3.4. Initial Screening of Ionic Liquids

With the purpose of initial screening, experiments were conducted at the mass ratio of ionic liquids and model solutions equal to 0.25. After phase separation, the compositions of model solutions were determined by means of gas chromatography. All investigated ionic liquids satisfy the major requirement during selection of solvent. In other words, all of them are insoluble in model solutions. These results are in concordance with the previously reported data [7] obtained with the same model solutions when [C_2_mim][EtSO_4_] and [C_6_mmPy] [Tf_2_N] were used as selective solvents.

**Figure 3:**
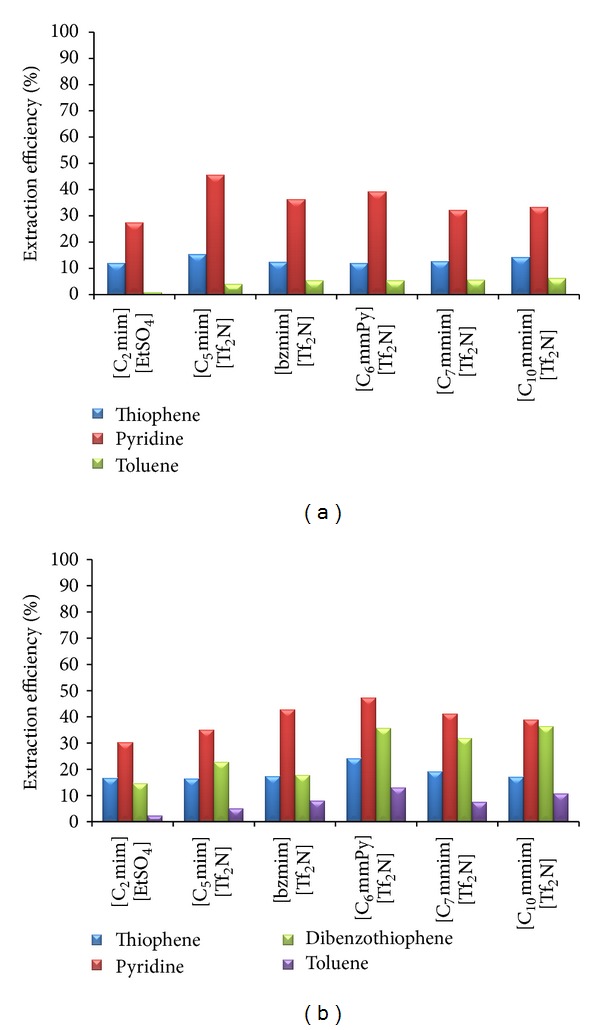
Initial screening of ionic liquids: (a) model solution 1; (b) model solution 2.

For the model solution that represents the FCC gasoline (model solution 1), results are presented in Figure 3(a). Since one of the most important conditions that solvent must fulfill is selectivity, other components of model solution should not be extracted with the selected ionic liquid. *n*-Hexane, *n*-isooctane, and *n*-heptane are insoluble in all investigated ionic liquids. On the other hand, toluene, as a representative of the aromatic hydrocarbons in model solution, is soluble in all ionic liquids except in [C_2_mim][EtSO_4_]. Partial dearomatization is favorable [21], so this ionic liquid is the least suitable ionic liquid. Basically, extraction efficiency of all ionic liquids is higher for pyridine than for thiophene. The influence of the length of the alkyl chain can be observed for the poly substituted imidazolium-based ionic liquids with long alky chain ([C_7_mmim][Tf_2_N] and [C_10_mmim][Tf_2_N]). Higher alkyl chain results in slightly higher extraction efficiency. Obtained result is in concordance with results obtained by other researchers that investigated the influence of the length of alkyl chain on the desulfurization efficiency of extraction of thiophene and pyridine from model fuels [2, 13, 14]. Extraction efficiency of all investigated ionic liquids for thiophene has a similar numerical value. Based on the obtained results, for denitrification and desulfurization of this model solution, the most suitable ionic liquid is [C_5_mim][Tf_2_N]. This ionic liquid has the lowest interfacial tension and viscosity (Table 3), so favorable conditions for mass transfer were achieved.

For the model solution that represents the diesel fuel (model solution 2), extraction efficiency of all investigated ionic liquids is shown in Figure 3(b). *n*-Heptane, *n*-dodecane, and *n*-hexadecane are insoluble in all investigated ionic liquids, so requirement concerning the selectivity was fulfilled. The majority of the investigated ionic liquids have higher affinity to thiophene and pyridine in the model diesel solution in comparison to the results obtained with gasoline model solution. Zhang et al. [9] observed quite opposite results with a set of different ionic liquids. Obviously, cation structure, selected anion, and its mutual interaction as well as interactions between compounds of the feed and the charged ion pairs of the ionic liquid influence the extraction capacity of ionic liquid. The extraction of dibenzothiophene is more difficult than extraction of thiophene and pyridine due to its complex structure, so this should be the major criteria for the selection of the most suitable ionic liquid. Having in mind that partial dearomatization is favorable, and ionic liquid selectivity to the key components (S- and N-compounds) [C_6_mmPy][Tf_2_N] can be the best choice. This ionic liquid shows the highest extraction efficiency to all key components. Poly substituted ionic liquids with long alkyl chain show high extraction capacity to dibenzothiophene. The usage of these two ionic liquids ([C_7_mmim][Tf_2_N] and [C_10_mmim][Tf_2_N]) should be further investigated from the ecological and toxicological points of view, because it was observed that toxicity is higher for longer alkyl chain substituents [25]. 

With this model solution, no general conclusions concerning the physical properties of the ionic liquids can be drawn. Different results can be found in the literature. For instance, Cer$\overset{´}{\text{o}}$n et al. [20] have reported that lower-viscosity ionic liquid is more effective for denitrogenation of real diesel. The authors have discussed their results also with different types of substituents on the pyridinium and imidazolium rings. Wilfred et al. [5] have investigated the influence of different process conditions and types of ionic liquids on the extraction efficiency of dibenzothiophene from dodecane. Based on their results, extraction efficiency increases with decrease of ionic liquid density. Both statements hold, but as previously mentioned many factors influence the mass transfer, not only physical properties. 

### 3.5. The Influence of (Ionic Liquid/Model Solution) Mass Ratio

**Figure 4:**
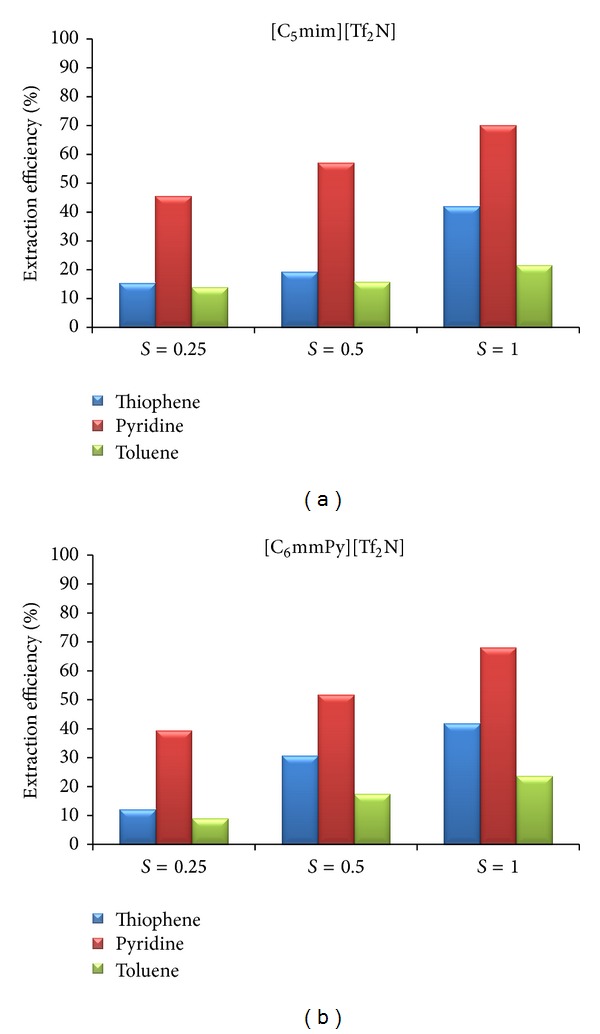
The influence of the mass ratio (ionic liquid/model solution) on the extraction efficiency of selected ionic liquids.

**Figure 5:**
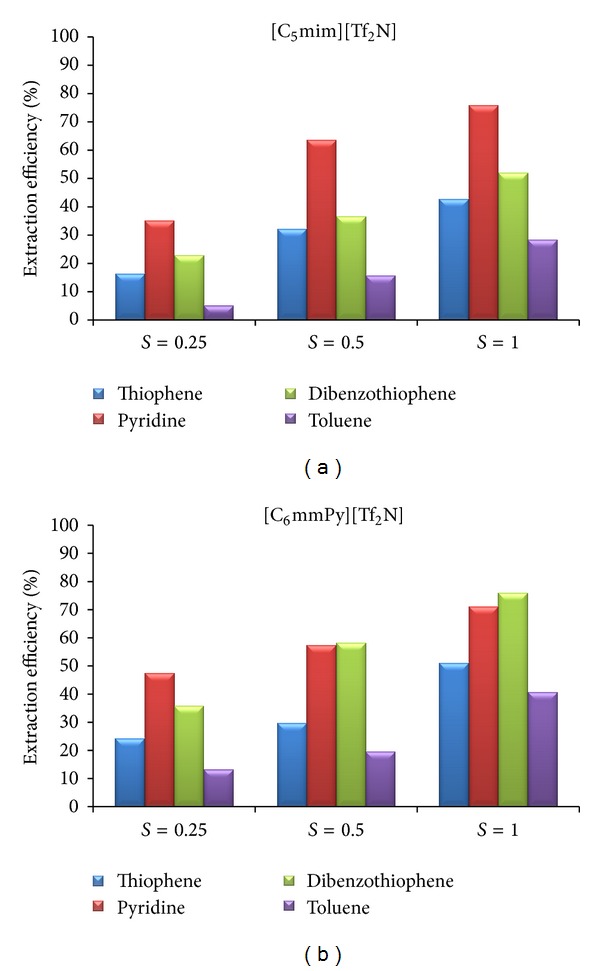
The influence of the mass ratio (ionic liquid/model solution) on the extraction efficiency of selected ionic liquids.

The influence of the mass ratio of ionic liquid to model solution on the extraction efficiency is presented in Figures 4 and 5. For both model solutions, results obtained with [C_5_mim][Tf_2_N] and [C_6_mmPy][Tf_2_N] are presented graphically, while the other results are commented.

#### 3.5.1. System Model FCC-Ionic Liquid

Higher mass ratio results in more efficient extraction of all soluble components. This influence was more pronounced for thiophene and pyridine than for toluene. For the system model FCC-[C_2_mim][EtSO_4_], extraction efficiency to thiophene was increased up to 26.7% and to pyridine up to 41.0%, when mass ratio, *S*, was increased from 0.25 to 1.00. For the system model FCC-[bzmim][Tf_2_N], extraction efficiency to thiophene was increased up to 17.7% and to pyridine up to 53.3%. When [C_7_mmim][Tf_2_N] was used, extraction efficiency to thiophene was increased up to 21.4% and to pyridine up to 50.8%. From the financial point of view, small mass ratio can be justified. Higher mass ratio means higher quantity of ionic liquid during extraction and finally higher amount of energy used for regeneration.

#### 3.5.2. System Model Diesel-Ionic Liquid

For the system model diesel-ionic liquid, similar results were obtained. Higher mass ratio positively influences extraction efficiency of all ionic liquids for the key components (Figure 5). Extraction efficiency at mass ratio, *S* = 1.0 for all other ionic liquids, is as follows: [C_2_mim][EtSO_4_] (thiophene: 40.3%; pyridine: 56.9%; dibenzothiophene: 37.8%); [bzmim][Tf_2_N] (thiophene: 43.9%; pyridine: 73.8%; dibenzothiophene: 48.3%); [C_7_mmim][Tf_2_N] (thiophene: 21.4%; pyridine: 50.8%; dibenzothiophene: 33.7%); [C_10_mmim][Tf_2_N] (thiophene: 50.8%; pyridine: 78.8%; dibenzothiophene: 67.1%).

### 3.6. Regeneration

**Figure 6:**
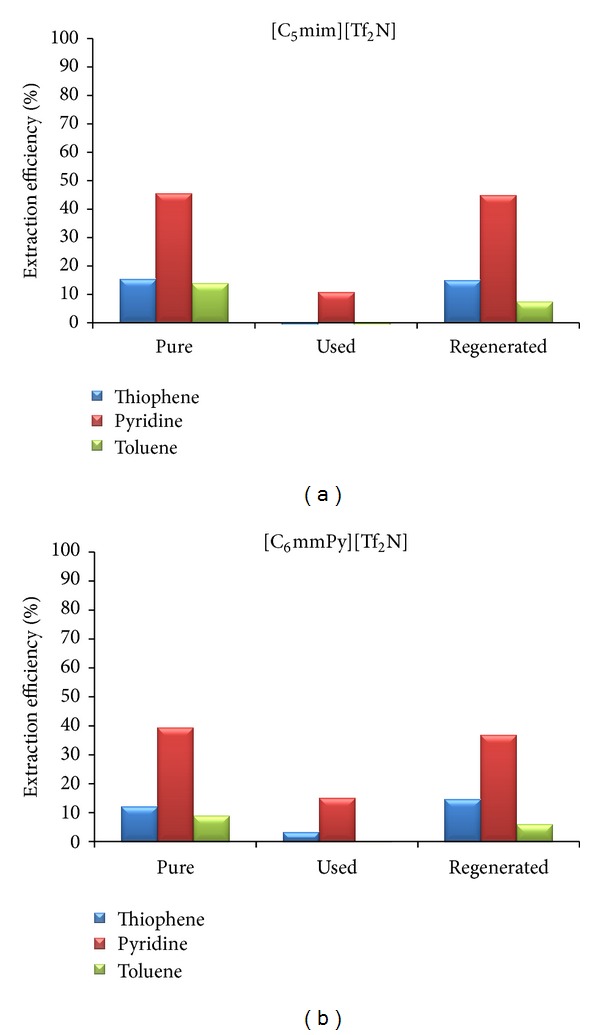
The influence of the solvent purity on the extraction efficiency (FCC model solution).

According to the literature, ionic liquids can be easily regenerated [5, 13–15, 18] and even used several times without regeneration [5]. For that reason, extraction was performed with contaminated and regenerated ionic liquids at the lowest mass ratio and previously determined mixing time. Obtained results for two selected ionic liquids are presented in Figures 6 and 8.

**Figure 7:**
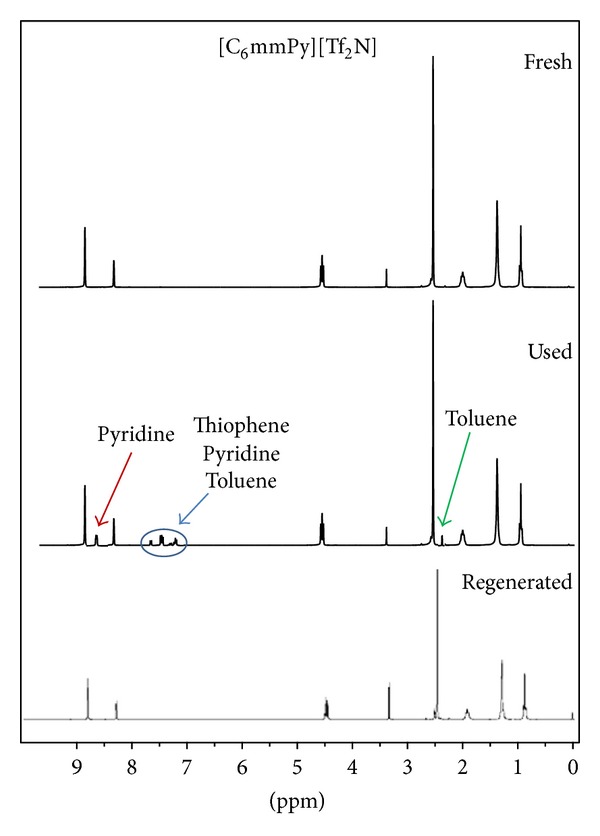
^1^H NMR spectra of fresh and used ionic liquids with model FCC solution and regenerated ionic liquids [C_6_mmPy][Tf_2_N].

One of the most important properties of ionic liquids, its nonvolatility, was used for regeneration. After extraction, contaminated ionic liquids were first used for another experiment and then purified by means of vacuum evaporation. For the model solution that represents FCC gasoline, obtained results are shown in Figure 6. When contaminated ionic liquid was used, extraction efficiency was reduced. Thiophene and toluene were not transferred to the ionic liquid, probably due to the saturation of ionic liquid with these two components during first usage. After regeneration, ionic liquids were totally purified, since reduction of the extraction efficiency was not observed. ^1^H NMR spectra (Figure 7) are the same for both fresh and regenerated ionic liquids. Peaks that belong to pyridine, thiophene, and toluene are visible on the used (contaminated) ionic liquid spectra.

**Figure 8:**
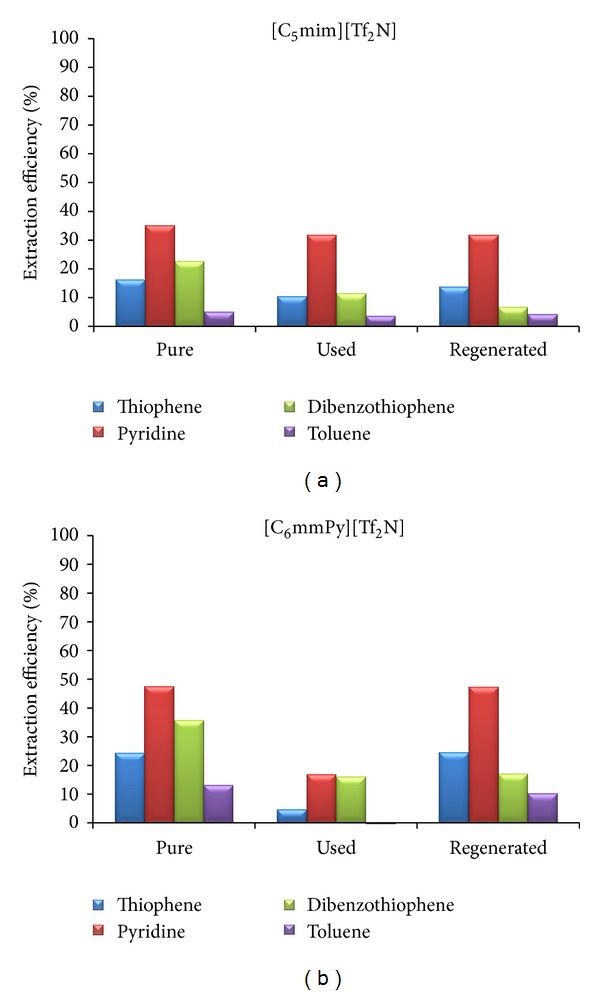
The influence of the solvent purity on the extraction efficiency (diesel model solution).

**Figure 9:**
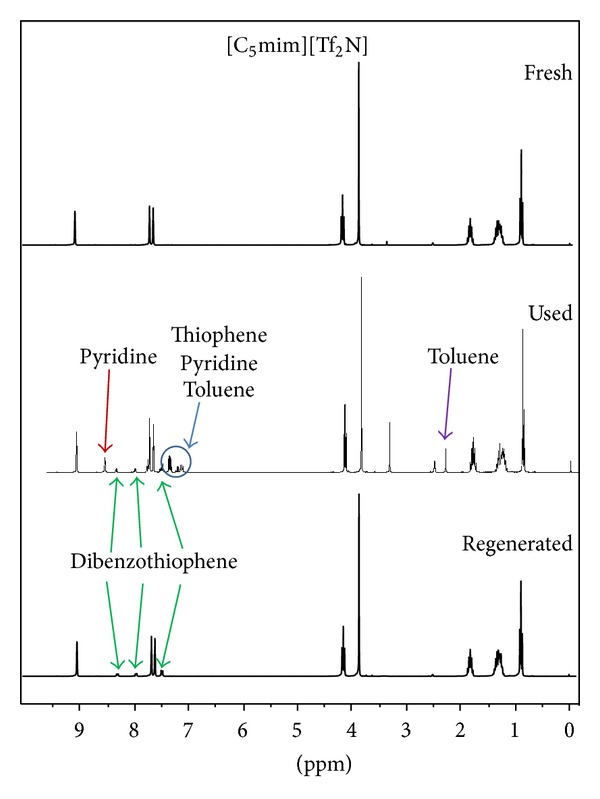
^1^H NMR spectra of fresh and used ionic liquids with model diesel solution and regenerated ionic liquids [C_5_mim][Tf_2_N].

Extraction efficiency of the regenerated ionic liquids for desulfurization and denitrification of model diesel solution was decreased (Figure 8). In a way, this result was expected since the boiling point of dibenzothiophene is very high (332°C). Therefore, dibenzothiophene cannot be removed by vacuum evaporation, so extraction capacity of ionic liquids is reduced. ^1^H NMR spectra of fresh, contaminated, and regenerated [C_5_mim][Tf_2_N] are shown in Figure 9.

Dibenzothiophene can be observed on the regenerated ionic liquid spectra. Dibenzothiophene can be removed by extraction with some suitable solvent, but in that way one additional regenerating step must be involved. As a consequence, the process will become economically and ecologically adverse. If ionic liquid is reused without regeneration, extraction efficiency for dibenzothiophene is drastically reduced, probably due to the saturation of ionic liquid with dibenzothiophene. This is an unwanted effect, because dibenzothiophene presents the major problem in most desulfurization processes [26].

### 3.7. Multistage Extraction

Multistage extraction has been performed with selected solvents for model FCC and model diesel solutions. Obtained results are presented in Table 4. Experiments have been performed at the lowest examined mass-ratio (*S* = 0.25) and short mixing time (*t* = 20 min). Four-stage extractions were performed, with 20-minute interval of phase separation between each stage. As expected, by increasing the number of extraction stages, mass fractions of thiophene and pyridine in the model FCC solution were decreased.

For the second group of the investigated system, model diesel-ionic liquid, extraction efficiency for all three key components is increased with increasing number of extraction stages. The highest efficiency was obtained with [C_6_mmPy][Tf_2_N] as the selective solvent.

If results obtained with the highest investigated mass ratio (*S* = 1) are compared with results obtained after four stages, when the same amount of ionic liquid was used, it can be concluded that multistage extraction is more efficient. In multistage extraction, after each stage fresh ionic liquid was used and mixed with the feed with lower concentrations of the key components. In that way, the driving force for mass transfer was increased, so higher amount of key component was transferred between phases.

## 4. Conclusions

Among six tested ionic liquids, [C_6_mmPy][Tf_2_N] is found to be the most effective selective solvent for desulfurizationa and denitrification of model solution that represents diesel at room temperature. For the other investigated model solution, [C_5_mim][Tf_2_N] is the most appropriate selective solvent. The extraction should be carried out at the lower mass ratio, to reduce amount of ionic liquid and consequently reduce energy used for regeneration. Ionic liquids can be successfully regenerated by means of vacuum evaporation. Regeneration was incomplete if model solution contains dibenzothiophene. If very low concentration of S- and N-compounds must be achieved, multistage extraction is the preferred choice.

It is necessary to reach an agreement between the defined purity and quality of gasoline and diesel fuel, by careful selection of the process conditions having in mind ecological and economical issues. 

Experiments with real FCC gasoline and diesel fuel should be performed in order to see whether the selected ionic liquid can be used for desulfurization and denitrification. The results with real samples cannot be predicted because real fuels consist of large number of compounds that can influence extraction efficiency and mutual solubility of ionic liquid and fuel.

## Supplementary Material

The GC program parameters for the analysis of both model solutions are presented in the following table.

## Figures and Tables

**Table 1:** Composition of the model fuels.

Model 1 (FCC gasoline)	Model 2 (diesel)
26% *n*-hexane 6% thiophene 26% isooctane 26% *n*-heptane 6% pyridine 10% toluene	3% thiophene 26% heptanes 6% pyridine 10% toluene 26% *n*-dodecane 26% *n*-hexadecane 3% dibenzothiophene

**Table 2:** Structures of selected ionic liquids.

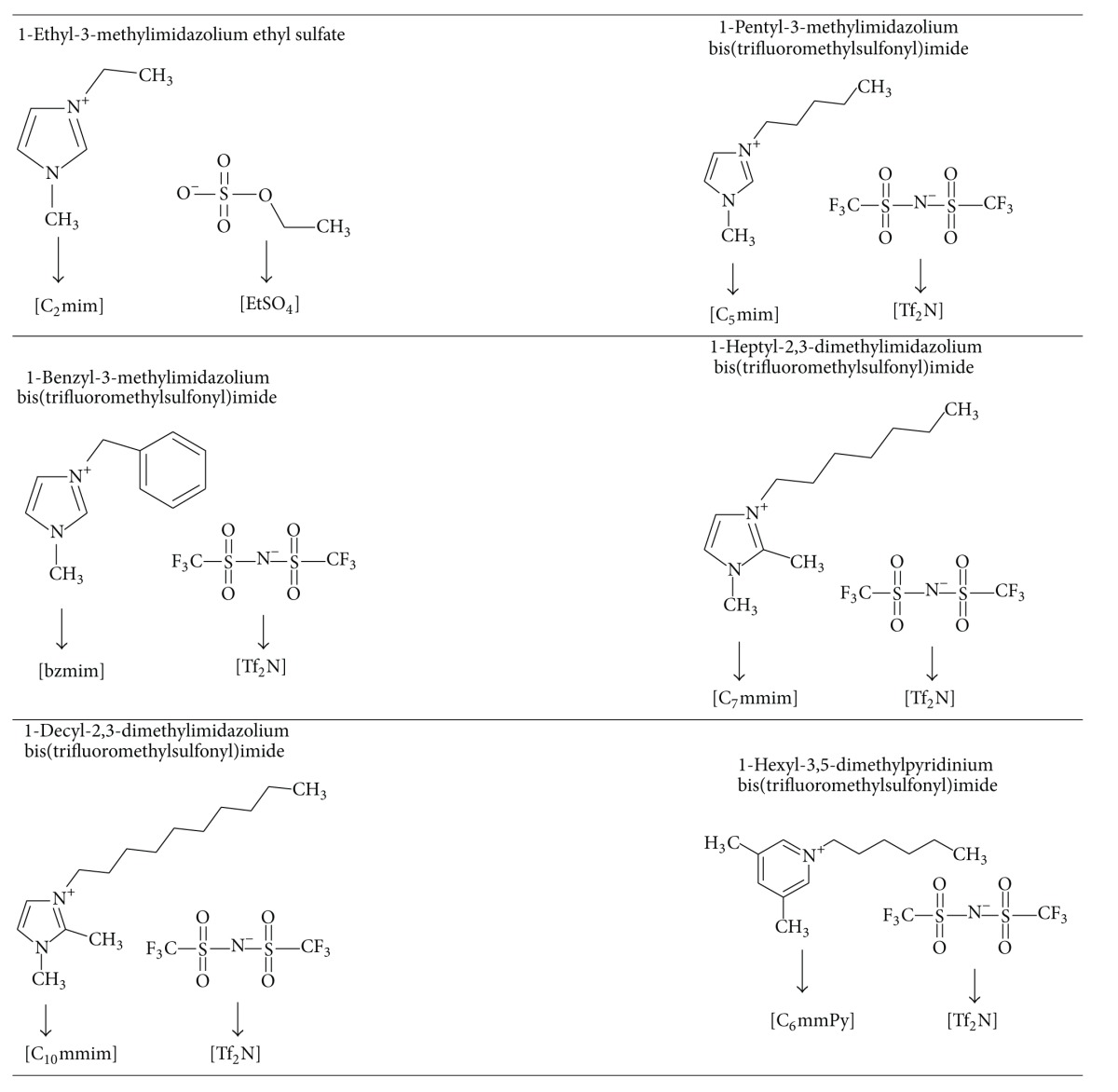

**Table 3:** Physical properties of model solutions and ionic liquids.

	*ρ*, kg m^−3^	*η*, Pa s	*σ*, mN m^−1^	*σ*_ 1-2_, mN m^−1^	
				Model 1	Model 2
Model 1	720	4.56 · 10^−4^	21.3		
Model 2	741	1.47 · 10^−3^	24.9		
[C_2_mim][EtSO_4_]	1236	0.0896	49.3	10.6	12.3
[C_5_mim][Tf_2_N]	1404	0.0525	32.6	3.2	5.6
[C_6_mmPy][Tf_2_N]	1332	0.1152	34.7	3.8	9.7
[bzmim][Tf_2_N]	1491	0.1508	41.5	6.0	6.2
[C_7_mmim][Tf_2_N]	1226	0.1048	46.9	10.5	12.1
[C_10_mmim][Tf_2_N]	1269	0.1472	33.3	3.4	5.8

**Table 4:** Extraction efficiency for multistage extraction of S- and N-compounds from the model solutions.

Ionic liquid	Component	Extraction efficiency%							
		FCC model solution				Diesel model solution			
		stage				stage			
		1	2	3	4	1	2	3	4
[C_2_mim][EtSO_4_]	Tiophene	12.01	24.11	32.98	39.85	16.60	25.66	39.94	49.36
	Pyridine	27.52	49.13	70.58	70.58	30.22	45.42	66.16	86.22
	DBT	—	—	—	—	14.70	25.14	33.32	42.40

[C_5_mim][Tf_2_N]	Tiophene	15.37	26.36	35.93	45.92	16.41	32.44	45.82	53.58
	Pyridine	45.62	58.63	70.18	78.59	35.12	49.58	76.93	87.80
	DBT	—	—	—	—	22.78	41.98	54.42	66.29

[C_6_mmPy][Tf_2_N]	Tiophene	12.05	24.71	40.06	50.53	24.21	29.53	43.08	65.59
	Pyridine	39.21	45.82	65.76	75.69	47.43	58.74	71.59	98.87
	DBT	—	—	—	—	35.67	59.10	71.38	92.61

[bzmim][Tf_2_N]	Tiophene	12.44	25.06	34.34	39.45	17.28	30.98	39.89	51.05
	Pyridine	36.19	54.72	68.37	70.17	42.82	62.59	72.59	80.57
	DBT	—	—	—	—	17.71	36.44	45.83	54.31

[C_7_mmim][Tf_2_N]	Tiophene	12.73	26.15	35.29	40.45	19.27	32.19	37.06	45.87
	Pyridine	32.16	53.27	67.60	70.64	41.08	65.57	78.67	81.04
	DBT	—	—	—	—	31.89	53.74	59.08	70.37

[C_10_mmim][Tf_2_N]	Tiophene	17.16	29.67	40.95	50.49	17.16	31.82	45.93	57.48
	Pyridine	32.48	54.36	66.98	77.11	38.85	60.43	74.08	81.51
	DBT	—	—	—	—	36.27	56.36	69.72	77.19

## Notations

*m*: Mass, kg
*S*: Mass ratio (ionic liquid/model solution)
*t*: Time, min
*x*: Mass fraction, %
*ε*: Extraction efficiency, %.

### Indices

*F*: Feed
*R*: Raffinate.
