# Supplementary material for: Extraction of S- and N-Compounds from the Mixture of Hydrocarbons by Ionic Liquids as Selective Solvents
Source: ScientificWorldJournal. 2013 Jun 12;2013:512953. doi: 10.1155/2013/512953 (PMC3694493; doi:10.1155/2013/512953)
Supplement: Supplementary file 1 — The GC program parameters for the analysis of both model solutions are presented in the following table. [file 512953.f1.docx]

GC conditions for determination of concentration

|  | Model 1 | Model 2 |
| --- | --- | --- |
| Carrier gas  Flow rate  Detector temperature  Injector temperature  Heating regime  Total analysis time | N_2_/air  2.5 mL/min  250 °C  100 °C  33 🡪70 °C (3 °C/min)  70 🡪 120 °C (30 °C/min)  18 min | N_2_/air  2.5 mL/min  250 °C  100 °C  70 🡪100 °C (10 °C/min)  100 🡪 250 °C (20 °C/min)  250 🡪 300 °C (25 °C/min)  17 min 50 s |
